# Evolutionarily conserved genetic interactions with budding and fission yeast *MutS* identify orthologous relationships in mismatch repair-deficient cancer cells

**DOI:** 10.1186/s13073-014-0068-4

**Published:** 2014-09-17

**Authors:** Elena Tosti, Joseph A Katakowski, Sonja Schaetzlein, Hyun-Soo Kim, Colm J Ryan, Michael Shales, Assen Roguev, Nevan J Krogan, Deborah Palliser, Michael-Christopher Keogh, Winfried Edelmann

**Affiliations:** Department of Cell Biology, Albert Einstein College of Medicine, New York, USA; Department of Microbiology & Immunology, Albert Einstein College of Medicine, New York, USA; Department of Cellular & Molecular Pharmacology, UCSF, San Francisco, USA; California Institute for Quantitative Biosciences, San Francisco, USA; School of Medicine and Medical Science, University College Dublin, Dublin, Ireland; J. David Gladstone Institutes, San Francisco, USA

## Abstract

**Background:**

The evolutionarily conserved DNA mismatch repair (MMR) system corrects base-substitution and insertion-deletion mutations generated during erroneous replication. The mutation or inactivation of many MMR factors strongly predisposes to cancer, where the resulting tumors often display resistance to standard chemotherapeutics. A new direction to develop targeted therapies is the harnessing of synthetic genetic interactions, where the simultaneous loss of two otherwise non-essential factors leads to reduced cell fitness or death. High-throughput screening in human cells to directly identify such interactors for disease-relevant genes is now widespread, but often requires extensive case-by-case optimization. Here we asked if conserved genetic interactors (CGIs) with MMR genes from two evolutionary distant yeast species (*Saccharomyces cerevisiae* and *Schizosaccharomyzes pombe*) can predict orthologous genetic relationships in higher eukaryotes.

**Methods:**

High-throughput screening was used to identify genetic interaction profiles for the MutSα and MutSβ heterodimer subunits (*msh2*Δ, *msh3*Δ, *msh6*Δ) of fission yeast. Selected negative interactors with MutSβ (*msh2*Δ/*msh3*Δ) were directly analyzed in budding yeast, and the CGI with SUMO-protease Ulp2 further examined after RNA interference/drug treatment in MSH2-deficient and -proficient human cells.

**Results:**

This study identified distinct genetic profiles for MutSα and MutSβ, and supports a role for the latter in recombinatorial DNA repair. Approximately 28% of orthologous genetic interactions with *msh2*Δ/*msh3*Δ are conserved in both yeasts, a degree consistent with global trends across these species. Further, the CGI between budding/fission yeast *msh2* and SUMO-protease *Ulp2* is maintained in human cells (*MSH2*/*SENP6*), and enhanced by Olaparib, a PARP inhibitor that induces the accumulation of single-strand DNA breaks. This identifies SENP6 as a promising new target for the treatment of MMR-deficient cancers.

**Conclusion:**

Our findings demonstrate the utility of employing evolutionary distance in tractable lower eukaryotes to predict orthologous genetic relationships in higher eukaryotes. Moreover, we provide novel insights into the genome maintenance functions of a critical DNA repair complex and propose a promising targeted treatment for MMR deficient tumors.

**Electronic supplementary material:**

The online version of this article (doi:10.1186/s13073-014-0068-4) contains supplementary material, which is available to authorized users.

## Background

**Figure 1:**
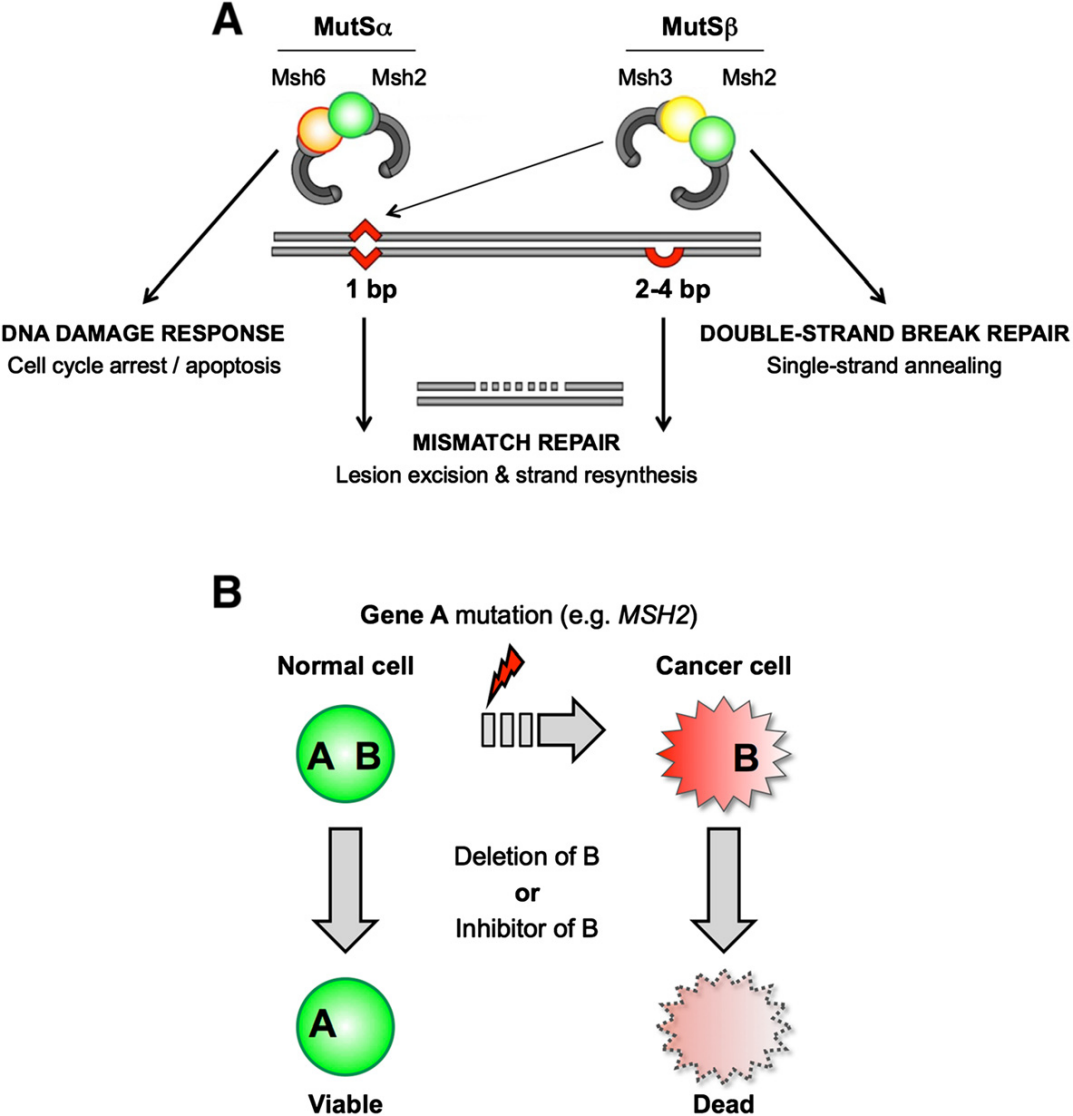
**Synthetic lethality as a therapeutic strategy for the treatment of MMR-deficient cancers. (A)** DNA mismatch repair is a stepwise process. In the schematic the MutSα heterodimer (Msh2-Msh6) recognizes a single-base mispair while MutSβ (Msh2-Msh3) primarily (but not exclusively [6-9]) initiates the repair of larger insertions/deletions (IDLs) of two to four bases. Recognition activates the recruitment of MutLα (Mlh1-Pms2) and multiple downstream factors (for example, Exo1, PCNA, RFC, RPA, and DNA pol δ) for repair by lesion excision and strand resynthesis. Each MutS also has roles independent of these downstream effectors of MMR: as an example, MutSα associates with cell cycle regulators at sites of DNA damage to mediate arrest and apoptosis [3], while MutSβ contributes to the repair of double-strand breaks via recombinatorial single-strand annealing [10,11]. Mutations in many factors from this pathway are associated with cancer. **(B)** Loss of A (for example, *MSH2*) is a common initiating event in many colorectal cancers. Candidate screening seeks to identify and exploit negative genetic relationships (synthetic sick/lethal) to selectively kill cells that harbor the *MSH2* deficiency, but spare those lacking this cancer-related alteration.

Defective DNA mismatch repair (MMR) is the underlying cause of hereditary non-polyposis colorectal cancer/Lynch syndrome (HNPCC/LS) and a significant proportion of sporadic colorectal cancers (CRCs) [1,2]. The MMR genes most frequently mutated or epigentically silenced in these cancers are MSH2 and MLH1, which respectively function in the coordination of mismatch recognition and excision [3,4]. The characteristic repair steps in MMR are highly conserved in bacteria, yeast, and mammals. In eukaryotes, the efficient recognition of distinct mismatches requires subsets of three different homologs of bacterial *mutS*: the MutSα heterodimer (MSH2-MSH6) initiates the repair of single-base mispairs and single-base insertion/deletions (IDLs), while MutSβ (MSH2-MSH3) primarily initiates the repair of larger IDLs of two to four bases but also facilitates the restoration of single-base mismatches [5] (Figure 1A). Subsequent to mismatch recognition MutSα or MutSβ interact with MutLα (MLH1-PMS2) in an ATP-dependent manner to initiate excision of the appropriate DNA strand [3]. During this process individual MutS and MutL subunits directly interact with PCNA, RFC, and RPA, indicating that mismatch excision is closely correlated with DNA replication [3].

As a consequence of their defective MMR, HNPCC/LS tumors and sporadic CRCs display increased rates of replication errors at short repeat sequences, termed microsatellite instability (MSI). MSI-positive tumors exhibit resistance to DNA damaging agents, and thus respond poorly to conventional chemotherapy [12,13]. Of additional concern, the treatment of these patients with chemotherapeutic agents can induce secondary therapy-related leukemias (for example, acute myeloid leukemia/myelodysplastic syndrome) [14,15]. Indeed it has been suggested that the primary treatment of HNPCC/LS with chemotherapeutics actually selects for hematopoietic precursor cells with MMR-defects [16]. As these cells proliferate they accumulate further mutations and develop increased resistance to anticancer agents. Thus the development of novel therapeutic strategies that efficiently and selectively target the primary MMR-deficient cancer but avoids therapy-induced secondary tumors would be highly desirable.

A promising new direction to develop targeted therapies is the harnessing of synthetic genetic interactions, where the simultaneous loss of two otherwise non-essential factors leads to reduced cell fitness (synthetic sickness (SS)) or cell death (synthetic lethality (SL)) [17,18]. This provides great functional insight: an SS/SL interaction for two alleles often indicates that their gene products are in parallel pathways or impinge on the same essential function. However it can also provide an elegant strategy to selectively eliminate tumor cells that harbor specific cancer-causing mutations. In this manner, the protein products of genes SS/SL to cancer-causing mutations represent potential drug targets (Figure 1B). As an example, inhibitors of poly-ADP-ribose polymerase I (PARP; required for the repair of single-stranded DNA breaks) are lethal to cells with deficiencies in *BRCA1* or *BRCA2* and show promise in the treatment of breast cancer [19,20].

A range of approaches can be used to search for SS/SL interactions with therapeutic intent, the most direct being RNA interference-based screening by si/shRNA libraries in human cells [21,22]. Although RNAi lends itself to high-throughput, incomplete knockdowns or off-target effects are commonly observed, and the large scale-format precludes single-case optimization. There are additional issues related to cost, that robotic sample handling might limit the type of read-out assay, the requirement for easily transfectable cell-lines, and even the possibility that an SS/SL interaction might be cell-type specific. Despite these potential concerns, various studies describe the successful application of high-throughput RNAi-based approaches to investigate mammalian gene function [21-24]. Indeed recent analyses identified the synthetic combinations *MSH2*/DNA pol β, *MLH1*/DNA pol γ, and *MSH2*/dihydrofolate reductase [25,26]. This directly demonstrates the existence of SS/SL interactions for mammalian MMR genes, although both DNA polymerases are essential in knockout mice and contribute to high-fidelity replication in dividing cells [27,28], which may limit their potential as therapeutic targets.

Genetic interaction screening in tractable model organisms constitutes a powerful alternative approach for candidate identification. Defining the function of a gene product in budding and fission yeasts (*Saccharomyces cerevisiae* (*Sc*) and *Schizosaccharomyces pombe* (*Sp*), respectively) has proven highly predictive of the role of its metazoan ortholog [29,30]. These yeasts separated approximately 380 million years ago [31] (by comparison, the last common predecessor of the entire mammalian class existed about 165 mya [32]), but share substantial gene content, with approximately 75% of *Sp* genes having one or more *Sc* orthologs [33-35]. This high level of conservation commonly extends to functional units, such that many yeast complexes are reminiscent of their metazoan counterparts [36,37]. High-throughput genetic interaction mapping approaches have been developed for both yeasts to evaluate the genetic interactions of null, hypomorphic, or mutant alleles in a genome-wide manner [33,34,38]. These techniques, in combination with the comprehensive deletion libraries available for both species [35,39], have been used to provide an overview of the functional dependencies within eukaryotic cells, and give insight into both the function of individual genes and the organization of biological systems [40-42]. Furthermore, cross species comparison has identified a high conservation (19% to 29%) of SS/SL interactions between orthologous gene pairs in *Sc* and *Sp* [33,34,41,42].

Here we surmised that complementary genetic analyses of specific MMR genes in the evolutionary distant *S. cerevisiae* and *S. pombe* could be used to dissect the function(s) of each individual factor. We further posited that screening against non-essential gene deletions could maximize the potential of identifying SS/SL interactions with potential therapeutic utility. Moreover the identification of Conserved Genetic Interactors (CGIs: SS/SL in both yeasts) could then be tested as candidate drug targets in MSI-positive CRC cell lines. An obvious potential limitation of this approach is the requirement for a high degree of conservation between the yeast and human orthologs. In this regard the MMR factors are particularly well conserved across evolution, which extends to preserving the same repair functions [43]: thus CGIs for each yeast gene might be expected to have strong predictive power for their mammalian orthologs.

## Methods

### *In situ* mutagenesis

Constructs for *de novo* gene deletion were assembled by PCR megapriming from budding or fission yeast genomic DNA and plasmid templates [44,45]. The resulting products were transformed/targeted by homologous recombination in the desired budding or fission yeast backgrounds and confirmed by sequencing and/or phenotypic analyses as appropriate [46].

### PEM2 (Pombe Epistatic Mapping) analyses in fission yeast

Genetic screening in fission yeast used the *PEM-2* approach [34,47]. In brief, NAT-marked (encoding nourseothricin resistance) queries in the *PEM-2* background (p392; KFP171) were crossed to a library of 1,955 non-essential gene deletions, with mating, haploid selection, data acquisition, and analysis as previously. Specific interactions of interest were further examined by direct mating, tetrad dissection and spot-testing. Pairwise correlation coefficients (CCs) to examine any relationship between genetic screens were calculated by the *CORREL* function in *Excel* [42].

### Approaches in saccharomyces cerevisiae

Many MMR factors participate in meiosis [48], such that some genetic interactions in fission yeast could be the result of a meiotic defect rather than manifesting during mitotic growth. To avoid this possibility genetic interactions in budding yeast were examined after direct transformation, plasmid shuffling by 5-fluoroorotic acid (5-FOA) selection, and spot-testing. In brief, specific deletions (as KAN cassettes flanked by approximately 500 bp of genomic sequence) were amplified from the relevant heterozygous diploid library strain (*Open Biosystems*), transformed to the appropriate *msh2*Δ or *msh3*Δ shuffle strain (with each NAT-marked genomic deletion covered by the relevant wild-type allele on a low-copy *URA3* containing plasmid), and homologous-integration events identified by PCR. Replicate clones were successively grown on media containing uracil (to allow loss of the *URA3* plasmid) and 5-FOA (converted to a toxic metabolite in the presence of a functional URA pathway, thus isolating double deletion cells). Spot-testing was then performed to evaluate the fitness of the double deletion clones versus their single deletion parents. Any sensitivity to genotoxins was investigated by spotting onto solid media with various concentrations of each agent: camptothecin (CPT) (5, 7.5, and 10 μM), methyl methanesulfonate (MMS) (0.005%, 0.075%, and 0.01%), hydroxyurea (HU) (5 mM, 7.5 mM, and 10 mM), or 5-fluorouracil (5-FU) (38 μM and 76 μM). Strains were spotted as 10-fold serial dilutions onto the relevant plates and growth examined at 48, 72, and 96 h.

### Approaches in mammalian cells

Standard methods were used to examine any genetic interactions in the *MSH2*-deficient human endometrial cancer cell line *HEC59* (*MSH2*^*−*^) and its isogenic chromosome 2-complemented counterpart (*MSH2*^*+*^) [49]. Transfections with siRNAs (Dharmacon; sequences in Additional file 1) and the *Trans*IT-siQUEST (Mirus) reagent were performed in six-well plates by specific optimization of the manufacturer’s suggested conditions. Total RNA was isolated by the *RNeasy plus* kit (Qiagen) 24 h after siRNA transfection, cDNA synthesized with random hexamers and the *Superscript III* reverse transcriptase (Invitrogen), and specific transcript levels measured by qPCR (after pre-amplification if required) [50]. SENP6 protein levels in permeabilized cells 48 h after siRNA transfection were measured by fluorescence cytometry after successive staining with monoclonal mouse anti-human SENP6 (Novus Biologicals) (or an isotype control antibody: eBioscience) and anti-mouse IgG2a-PE (eBioscience) (Additional file 1: Figure S1). Apoptosis in specific populations was quantified by activated caspase 3. In brief, cells were trypsinized, stained with *LIVE/DEAD fixable violet* (Invitrogen), fixed with 2% paraformaldehyde, and permeabilized with 0.3% saponin. Each sample was then successively stained with rabbit anti-caspase 3 (Cell Signaling Technology) and goat anti-rabbit IgG-Alexa488 (Molecular Probes), and analyzed by fluorescence cytometry. DNA double strand break levels in specific populations was quantified by γH2AX. In brief, 72 h after siRNA transfection/24 h after PARP inhibition (20 μM olaparib), cells were permeabilized with Transcription Factor Buffer (BD Pharmingen), successively stained with rabbit anti-γH2AX (Cell Signaling Technologies) and goat anti-rabbit IgG-Alexa488, and analyzed by fluorescence cytometry. To estimate clonogenic survival, cells were re-plated 72 h after siRNA transfection/24 h after PARP inhibition (5 μM olaparib). In brief, cells were collected after each treatment (of triplicate wells), resuspended in the same volume of media, diluted 1/500, and an equal aliquot from each sample re-plated and incubated for 2 weeks. Cells were fixed with 2% paraformaldehyde, stained with crystal violet, colonies counted, and the clonogenic potential after each treatment of each population expressed relative to the respective *siNT-1* (no drug).

## Results and discussion

For this study three specific MMR genes involved in mismatch recognition (*MSH2*, *MSH3*, and *MSH6*, comprising subunits of the MutSα and β heterodimers: Figure 1A) were chosen for direct analysis. *MSH3*^−/−^ mice have late onset intestinal tumors and MSH3-deficiency modifies the tumor spectrum of p53 mutant mice [10,51]. *MSH3* mutations are rarely seen in HNPCC/LS, although frame-shifts at repeat sequences within the gene are frequently detected in MSI positive CRCs [2,52,53]. *MSH2* is frequently mutated in HNPCC/LS, while *MSH6* mutations are more rare (although *MSH6*, like *MSH3*, is also commonly frame-shifted in MSI positive CRCs [52,54]). Thus the specific genetic interactors of each gene could identify novel therapeutic targets for these conditions. Comprehensive genetic interaction data for all three factors would also be expected to distinguish the relationship of MutSα and MutSβ with other repair pathways or biological processes.

### Genetic interaction profiling distinguishes fission yeast MutSα and MutSβ

**Figure 2:**
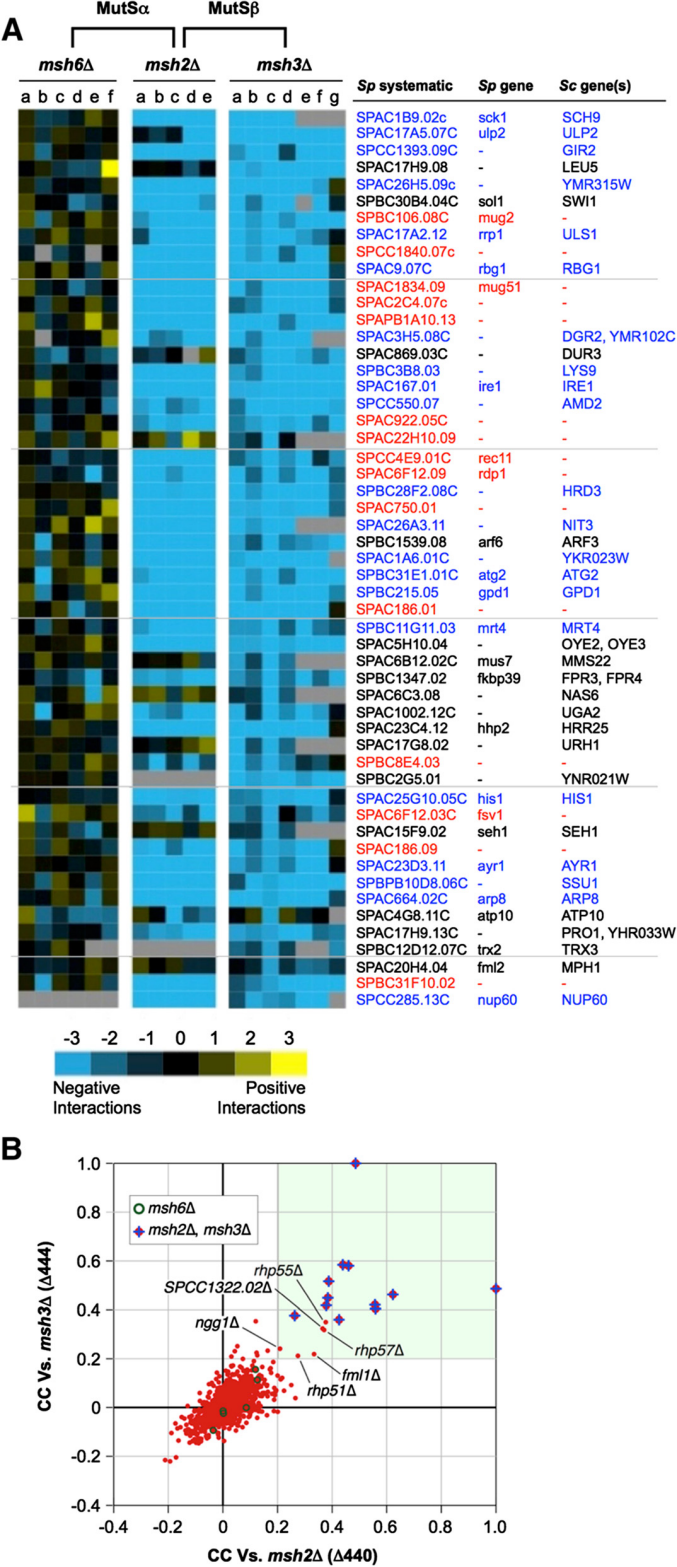
**Genetic screening distinguishes the MutSβ** **complex in actively growing yeast. (A)** The specific genetic interactions of fission yeast *msh2*Δ and *msh3*Δ are similar and distinct from *msh6*Δ. *NAT*-marked deletions (for example, *msh2*Δ::NAT) were placed in the context of 1,955 non-essential fission yeast gene deletions and relative colony size used to derive scores covering each negative (≤ −2.5) genetic interaction (see Methods). Shown are a representative set of 53 genetic relationships expressed by heat map (key below) and identified by their systematic ID and standard *Sp*/*Sc* (if present) gene names. Blue, orthologous relationships also tested in budding yeast (see Figure 3). Red, *Sp*-specific orphans. Given the high rate of replication-errors in MMR-gene mutants [56,58], multiple queries of each deletion were screened to obtain a high confidence dataset. The independent accumulation of confounding secondary mutations may explain why the specific genetic interactors from this study are not described in the available high-throughput datasets that relied on single deletion clones [33,40,59]. **(B)** Distribution plot of Pearson correlation co-efficients (CCs) comparing the genetic interaction profiles of *msh2*Δ and *msh3*Δ within a (953 × 1,955) dataset. Red dots indicate the CC of each genetic screen to a specific *msh2*Δ or *msh3*Δ query clone: shaded area highlights screens that would be classed as significantly correlated (CC ≥0.2).

Appropriate deletion strains (for example, *msh2*Δ::NAT) were mated to a library of 1,955 deletions (approximately 52% of the non-essential *Sp* genome [35]), and double-mutant haploid daughters selected with the *Pombe Epistatic Mapper-2* (*PEM-2*) approach [34,47] (see Methods). We then used colony size (compared in high-throughput by photography and image analysis) as a quantitative readout to derive scores covering each negative genetic interaction (< −2.5: SS/SL) [34,42]. Of note, the deletion of *msh3* leads to a modest increase in mutation rates [55], while *msh2*Δ or *msh6*Δ each reduce the fidelity of DNA replication >100-fold in *S. pombe* [56], *S. cerevisiae* [55] and mammals [57]. The resulting accumulation of secondary mutations had the potential to compromise our genetic screening, so multiple independent clones of each deletion were analyzed to obtain a high-quality dataset (Figure 2A). To date we have screened 953 query alleles against an array of 1,955 non-essential deletions and comprehensive benchmarking confirms the value of this resource: known SS/SL interactions are reproduced, and protein-protein interactions are accurately predicted [34,42]. Analysis of the *msh2*Δ, *msh3*Δ, and *msh6*Δ replicates confirm these global trends, with their data being highly correlated (Pearson correlation co-efficient (CC) >0.4) indicating that individual SS/SL predictions are highly reproducible (for example, Figure 2A and B).

The genetic interaction (GI) profile of a mutant allele comprises its set of interacting partners, or in the case of our quantitative screening, the set of scores for these interactions (for example, Figure 2A). GI profiles have been used to predict gene function with high confidence, while the degree of similarity to other mutant profiles can reveal how groups of gene products cooperate in higher-level biological processes [34,42,60,61]. As an example, Msh2 enters into heterodimeric complexes with Msh3 and Msh6, so it might be expected that the set of genetic interactions for *msh2*Δ would encompass those observed on deletion of both its partners. Alternatively, since MutSα (Msh2-Msh6) is sufficient for the majority of fission yeast MMR [56], the genetic interactions for *msh2*Δ and *msh6*Δ could have been very similar. To investigate this we compared the profile for each *msh*Δ within our (953 × 1,955) dataset [42], with significant relatedness considered a Pearson CC >0.3 [34,42,60]. In this approach *msh2*Δ had no discernible relationship to *msh6*Δ, but was instead highly correlated with *msh3*Δ (Figure 2B). We considered that *msh2* and *msh3* may be genetically related by their loss of function in a process other than MMR. Budding yeast MutSβ also acts with the Rad1-Rad10 nuclease to remove non-complementary tails during DNA DSB repair by homologous recombination [11] (Figure 1A). We thus noted with interest that the GI profiles of *msh2* and *msh3* were both highly correlated with those of *fml1* (*Sc* Mph1; ATP-dependent 3′ to 5′ DNA helicase, FANCM ortholog), *rhp55* and *rhp57* (both RecA family ATPases), and *rhp51* (*Sc* Rad51; RecA family recombinase) (Figure 2B), all of which regulate DNA double-strand break (DSB) repair by recombination [62,63]. Thus the similar GI profiles of fission yeast *msh2*Δ and *msh3*Δ might be due to their shared role in recombinatorial rather than mismatch repair [58,64,65].

Network analyses suggest that the average orthologous gene has significantly more genetic interactions than sequence orphans (that is, genes with no identifiable orthologs in other species) [42]. The *MSH* genes are highly conserved, yet *msh6*Δ showed an unexpectedly low percentage of negative GIs relative to all profiles in our (953 × 1,955) dataset (4.6% *msh2*Δ; 2.5% *msh3*Δ; 1.1% *msh6*Δ vs. 3.8% average [42]). This distinction from the general trend suggested a limited cross-talk between Msh6 and other pathways: this could be because its protein product acts exclusively in MMR, although such an interpretation is made with caution. In this study genetic interactions were revealed by the specific read-out of altered colony size on rich media. Repeating these analyses in the presence of genomic stress (for example, genotoxin-induced damage) might uncover additional, or an altered spectrum, of synthetic interactions [61,66], and thus reveal more functional relationships for MutSα.

### Identification of evolutionarily conserved GIs for *msh2*Δ/*msh3*Δ in fission/budding yeasts

We next sought evolutionarily conserved genetic interactions (CGIs) for fission and budding yeast *msh2*Δ/*msh3*Δ to identify orthologous relationships of possible utility in mammalian systems. Twenty-six candidates (25 one-to-one orthologs and the paralogous pair *mlp1*/*mlp2*) from the fission yeast SS/SL dataset (for example, Figure 2A) were chosen for direct study by satisfying three criteria: synthetic with both *msh2*Δ and *msh3*Δ; identifiable *Sc* and human/mouse orthologs (or a limited group of paralogs); and non-essential in both *Sc* and human/mouse, thus increasing their potential therapeutic utility. Each mutant combination was created and examined for growth on rich-media and any additional sensitivity to genotoxins, which included 5-fluorouracil (5-FU). This agent is a commonly used chemotherapeutic for colorectal cancer, although MMR-deficient MSI-positive tumors display resistance [12,13]: thus synthetic chemo-sensitization in any double-mutant background would be of particular interest.

**Figure 3:**
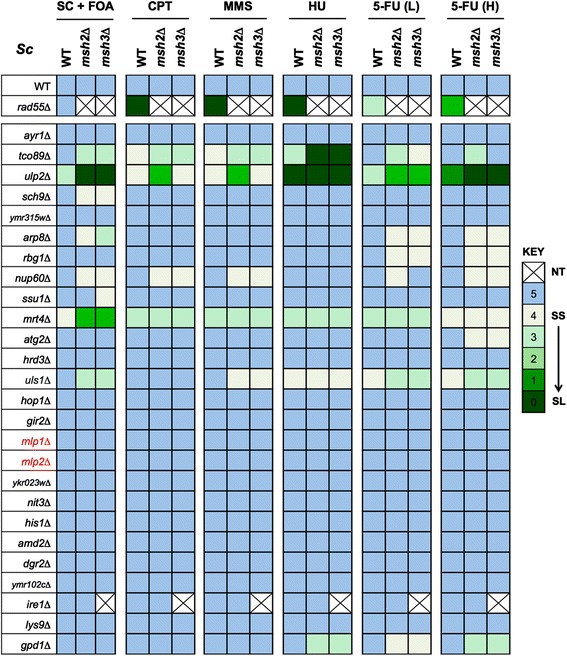
**Direct testing identifies conserved orthologous relationships with MutSβ** **in fission and budding yeast.** Negative interactions from fission yeast were tested for orthologous conservation and potential synthetic chemo-sensitivity in budding yeast. *msh2*Δ- or *msh3*Δ-containing strains were created by plasmid shuffling and spotted as serial 10-fold dilutions onto the indicated medium (see Methods). To facilitate cross-comparison results are expressed by a color-code indicating the growth of each strain on a six-point scale relative to wild-type in each condition (all after incubation at 30°C for 72 h). *rad55*Δ is a positive control for genotoxin sensitivity [74]. Hatch-bar, not tested; YPD, non-selective media; CPT (5 μM); MMS (0.005%); HU (5 mM); 5-FU (*L*ow dose, 38 μM); 5-FU (*H*igh dose, 76 μM).

Testing these 26 candidates identified seven orthologous negative interactions, with a genotoxin-aggravating effect on the double mutant observed in most cases (Figure 3). This moderate number of CGIs for *msh2*/*msh3* (7/25 or approximately 28%) is consistent with recent studies interrogating the conservation of global genetic interaction trends across both yeasts [41,42]. The large-scale studies had further noted that GIs within functionally related gene pairs (such as those involved in the same pathway or process) are more highly conserved across species than those between seemingly unrelated gene pairs [41,42]. However the specific CGIs for *msh2*Δ and *msh3*Δ (Figure 3) appear to be involved in quite diverse biological functions. Tco89, for example, is a subunit of the TORC1 complex that regulates growth in response to nutrient availability [67]; Sch9 is an AGC family protein kinase downstream of TORC1 mediated regulation of ribosome biogenesis, translation initiation and entry into G0 [68]; Nup60 is a nucleoporin component of the nuclear pore complex [69] and involved in gene tethering to the nuclear membrane; Arp8 is an actin-related protein and subunit of chromatin remodeling complexes [70]; and Mrt4 regulates mRNA turnover and ribosome assembly [71]. Although the molecular basis for these genetic interactions is currently unclear, their conservation across such evolutionary distance indicates functional importance and may reflect the diverse pathways involved in genome maintenance. The CGIs between *msh2*Δ/*msh3*Δ and *tco89*Δ, for example, may reflect the sensitivity of MMR-deficient colorectal cancer cells to mTOR inhibition by rapamycin [72]. Likewise the CGIs with *nup60*Δ may be linked to the role of the (Nup60/Mlp1-2) nuclear pore complex in maintaining SUMO-protease Ulp1 at the nuclear envelope to regulate the sumoylation of several proteins, including DNA repair factors [73].

**Figure 4:**
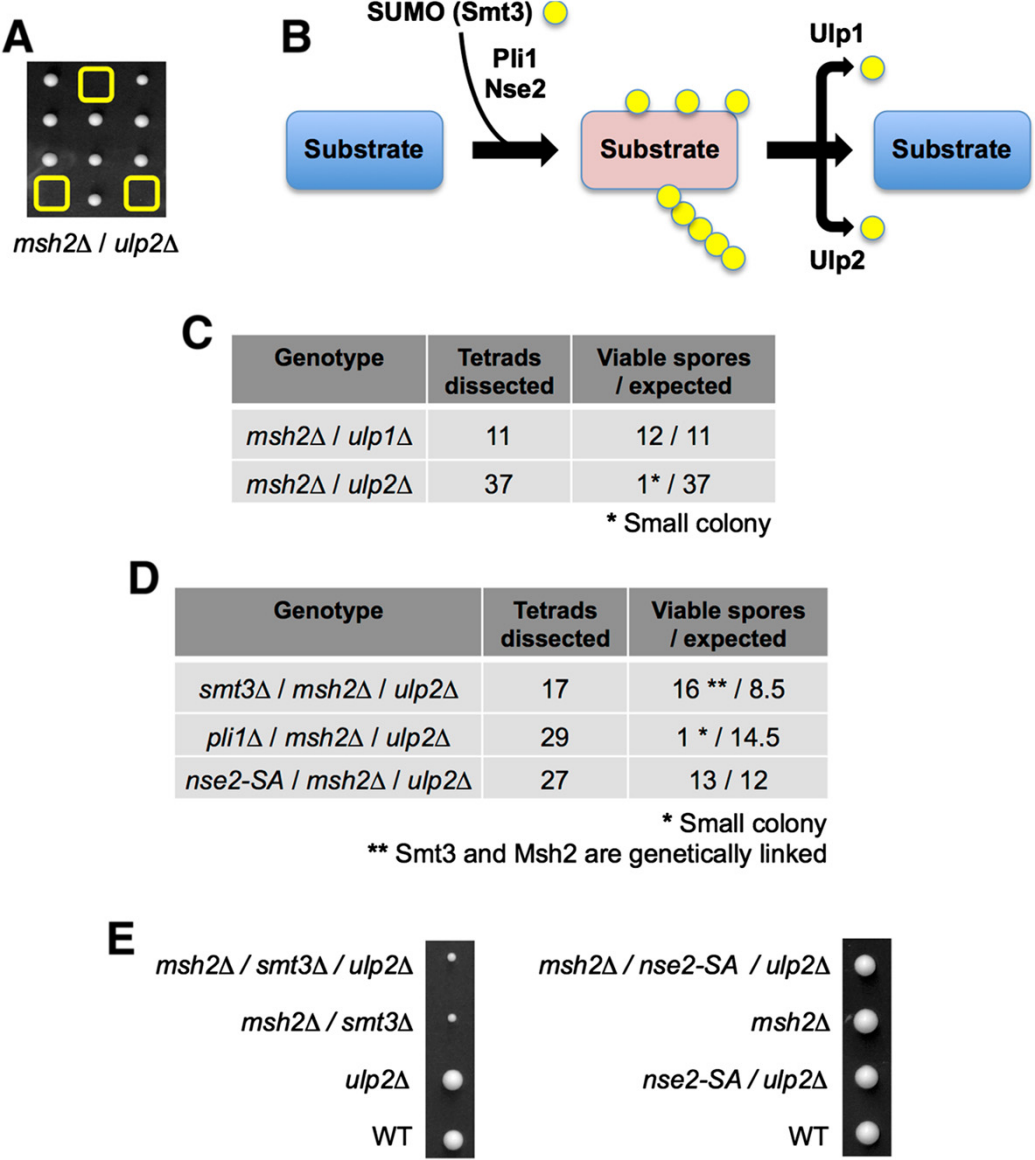
**Increased polysumoylation is lethal in** ***msh2***Δ **fission yeast. (A)** Deletion of the Ulp2 SUMO-protease is synthetic lethal (SL) with *msh2*Δ. *msh2*Δ and *ulp2*Δ strains were mated, sporulated, tetrads dissected and genotypes determined ((*ulp2*Δ/*msh2*Δ) is boxed). **(B)** Schematic depicts the sumoylation pathway. Fission yeast contains one gene encoding SUMO (Smt3), two E3-ligases (Pli1 and Nse2), and two SUMO proteases (Ulp1 and Ulp2). **(C)** *msh2*Δ is SL with *ulp2*Δ but neutral with *ulp1*Δ. Strains were mated, sporulated, and tetrads dissected. The number of viable spores observed/expected for each genotype is indicated (see also Additional file 1: Figure S2A and B). **(D, E)** *smt3*Δ or *nse2-SA* (catalytic dead), but not *pli1*Δ, rescue the lethality of (*msh2*Δ/*ulp2*Δ). Strains were mated, sporulated, and the indicated number of tetrads dissected (see also Additional file 1: Figure S2C to E). Panel **(E)** depicts representative tetrads to demonstrate the growth of each mutant combination.

The group of CGIs common to *msh2*Δ and *msh3*Δ contains two additional factors involved in protein sumoylation: Ulp2 (*Sc* Ulp2) and Rrp1 (*Sc* Uls1) (Figures 2A and 3). The Ulp2 peptidase deconjugates SUMO polychains from proteins and plays a role in the recovery from checkpoint arrest induced by DNA damage or replication defects [75,76]. The Rrp1 ATPase regulates the proteolytic control of sumoylated substrates and the response to replication stress [77,78]. This cluster of CGIs strongly suggested a ‘cross-talk’ between MutSβ and the SUMO pathway. The (*msh2*Δ/*ulp2*Δ) interaction is synthetic lethal in fission yeast (Figures 2 and 4A), synthetic sick and aggravated by 5FU (a commonly used chemotherapeutic agent) in budding yeast (Figure 3). We thus chose to perform a more detailed genetic analysis of this specific CGI in fission yeast before further testing the orthologous relationship in mammalian cells.

### Increased polysumoylation is toxic to *msh2*-deficient yeast cells

Sumoylation is a reversible post-translational modification that controls the localization, function, interaction, and stability of a large number of proteins, including many involved in transcription, replication, and the DNA damage response [79]. Indeed recent studies highlight the importance of a reversible SUMO-response to preserve genome integrity [80,81]. Fission yeast encodes one form of SUMO (Smt3 aka. Pmt3), two SUMO E3-ligases (Pli1 and Nse2), and two SUMO proteases (Ulp1 and Ulp2; Figure 4B). Pli1 catalyzes the majority of cellular sumoylation, while Nse2 targets a limited number of substrates during DNA repair [82,83]. For deconjugation, Ulp1 removes single SUMO modifications and is required for efficient cell cycle progression [84,85]. Ulp2, in contrast, edits poly-SUMO chains, desumoylates the majority of factors activated in response to DNA damage or DNA replication defects, and regulates the recovery from checkpoint arrest [75,76,86,87].

On direct testing the SL interaction of (*msh2*Δ/*ulp2*Δ) is not observed with (*msh2*Δ/*ulp1*Δ) (compare Figure 4A and C), indicating that the accumulation of specific sumoylated substrates is toxic to fission yeast cells that also lack an Msh2-mediated repair pathway. This may be because Msh2 is absolutely required to resolve lesions that occur at increased levels in the context of *ulp2*Δ [88]. Alternatively cells lacking Msh2 might accumulate unresolved DNA damage and induce a poly-sumoylated substrate (or substrates) that cannot be metabolized in the absence of Ulp2, blocking cell cycle progression [87]. The identity of such substrates is currently unknown, though many central players in replication and recombination (for example, Rad52, PCNA, RPA) are sumoylated in response to DNA damage, and this is important for their repair function [79]. To investigate this further we tried to create triple mutants containing *msh2*Δ, *ulp2*Δ and a mutation in either SUMO itself (*pmt3*Δ) or one of its E3-ligases (*pli1*Δ, or the *nse2-SA* allele that lacks SUMO ligase activity but retains the essential function for Nse2 in chromosome maintenance [89]). Following tetrad dissection we obtained the triple mutant combinations (*msh2*Δ/*ulp2*Δ/*pmt3*Δ) and (*msh2*Δ/*ulp2*Δ/*nse2-SA*), but not (*msh2*Δ/*ulp2*Δ/*pli1*Δ) (Figure 4D and E and Additional file 1: Figure S2). This clearly demonstrates that *msh2*Δ is lethal in combination with a deletion of Ulp2, the primary protease to desumoylate DNA damage response proteins [87,88], but this can be rescued by inactivating the primary ligase for sumoylation in response to DNA damage [82,83].

### Conservation of the GI between *MSH2* and the deSumoylation pathway in mammals

We next sought to examine if the CGI between budding and fission yeast *msh2* and *ulp2* predicted an orthologous relationship in human cells. Humans encode four SUMO proteins (SUMO1-4 with relatedness: SUMO1, SUMO2/3, unconjugated SUMO4) and six SUMO-specific cysteine proteases (SENPs). Of these, SENPs 1/2/3/5 are most closely related to Ulp1 and specifically deconjugate single SUMO1/2/3, while SENPs 6/7 resemble Ulp2 and edit polysumo chains with a clear preference for SUMO2/3 [90-92]. A limited literature discriminates the distinct roles of mammalian SENP6 and SENP7 [93-95]. However both are expressed in a variety of tissues including the colonic epithelium [90] and thus might be active in MMR-deficient colorectal cancer cells.

**Figure 5:**
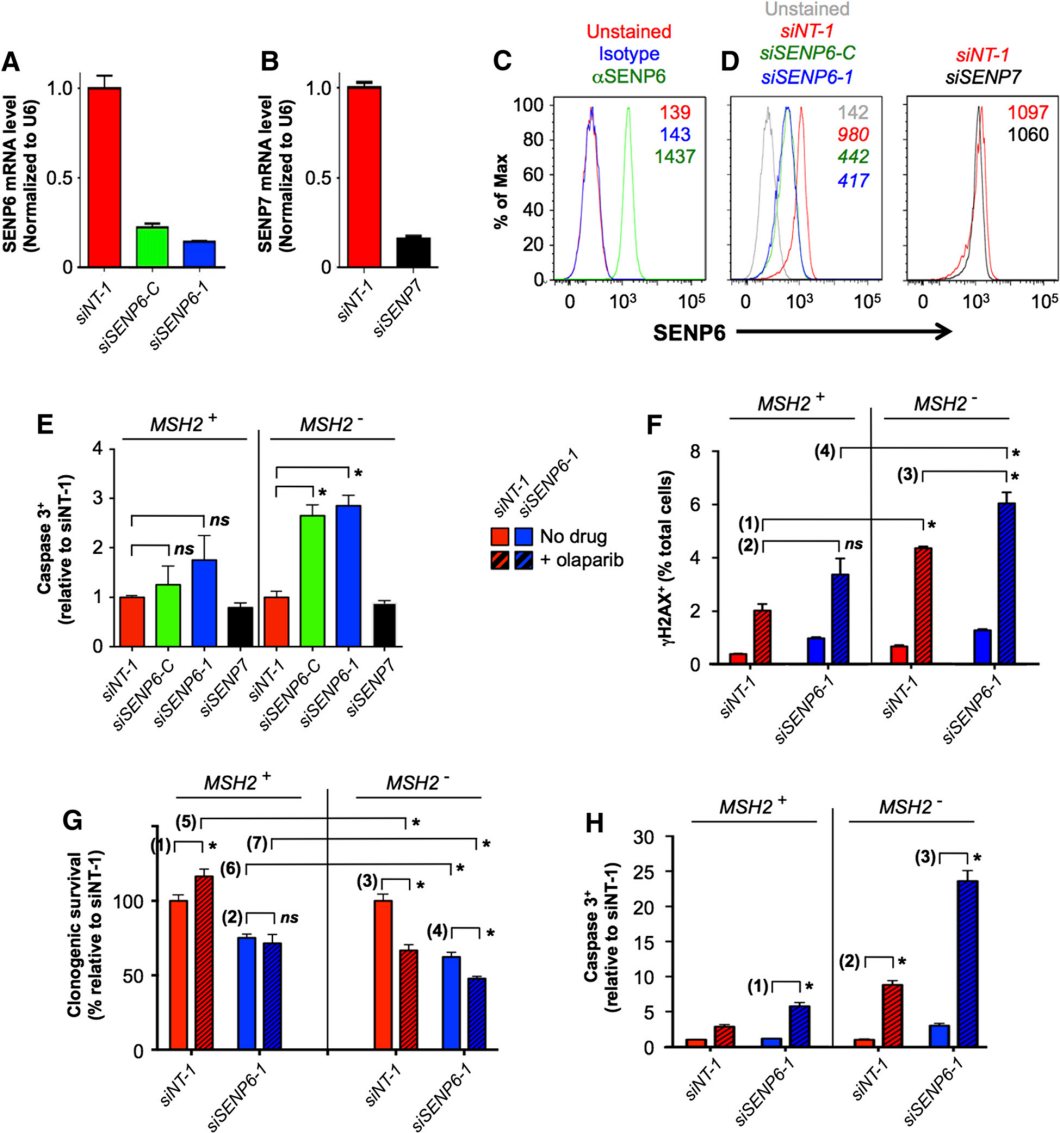
**SENP6 knockdown induces a hyper-acute apoptotic response in MSH2-deficient human cells. (A-D)** Efficient targeted knockdown of SENP6 or SENP7 in *MSH2*-deficient *HEC59* cells. In **A**, **B** mRNA was quantified by qPCR; in **C**, **D** SENP6 protein was detected by flow cytometry (see Methods). Note specificity of SENP6 knockdown in response to *siSENP6-C* or *siSENP6-1* [93], but not *siSENP7* (pooled *siSENP7-1* and *siSENP7-2*) or non-targeted *siNT-1*. **(E)** SENP6 (but not SENP7) knockdown induces apoptosis in *HEC59* cells (*MSH2* ^−^) relative to an isogenic chromosome 2 complemented population (*MSH2* ^+^). Caspase 3^+^ cells were identified 48 h after siRNA transfection (see Methods), normalized to *siNT-1* in each population, and significance determined by unpaired t-test (*ns*, not significant; *, *P* <0.01). **(F)** PARP inhibition induces DNA DSBs (γH2AX^+^ [97]), and to a greater degree in *MSH2* ^*−*^ cells. γH2AX^+^ cells were identified 72 h after siRNA transfection/24 h after PARP inhibition (20 μM olaparib; see Methods) and the significance of pairwise comparisons determined by unpaired t-test: (1) *P* <10^−4^; (2) *ns*, not significant; (3) *P* <0.008; (4) *P* <0.002. Panels **(F-H)** use the same color key and *siSENP6-1* for SENP6 knockdown (previous studies suggest off-target effects are unlikely [93]). **(G)** *MSH2* ^*−*^ cells show reduced clonogenic survival (see Methods) in response to *SENP6* knockdown, PARP inhibition, or their combination. Any significance of indicated pairwise combinations was determined by unpaired t-test: (1) *P* <0.02 (may be related to the additional copy of chromosome 2); (2) *ns*, not significant; (3) *P* <0.001; (4) *P* <0.01; (5) *P* <0.0007; (6) *P* <0.03; (7) *P* <0.01. **(H)** (*MSH2*/*SENP6*) deficient cells exhibit a hyper-apoptotic response to PARP inhibition. Activated caspase 3 levels 72 h after siRNA transfection/24 h after PARP inhibition (20 μM olaparib) were quantified by flow cytometry and normalized to *siNT-1* (no drug). Any significance of various pairwise combinations was determined by unpaired t-test: (1) *P* <10^−5^; (2) *P* <10^−6^ (also seen with *MSH3* ^*−*^ [98]); (3) *P* <10^−7^.

To investigate any relationship between human MSH2, SENP6, and SENP7 we used siRNA to efficiently knockdown each SUMO protease in *HEC59* cells (an Msh2-deficient endometrial cancer line) and their isogenic chromosome 2-complemented MSH2 proficient counterparts (Figure 5A to D). SENP6 knockdown resulted in significantly increased apoptosis in MSH2-deficient relative to MSH2-proficient cells (Figure 5E), reproducing the negative genetic interaction identified in budding and fission yeasts (Figures 2A and 3). Interestingly SENP7 knockdown had no effect (Figure 5E). This SUMO protease was recently shown to interact with chromatin remodelers and contribute to chromatin relaxation during DNA repair by homologous recombination [94-96]. However our results indicate that the effect of SENP7 knockdown on recombinational repair does not lead to increased apoptosis in *MSH2*-deficient cells.

Human SENP6 maintains the hypo-sumoylation of RPA70, and thus regulates homologous recombination-mediated repair during S-phase [93]. This finding allied with our results in yeast may imply that the conserved synthetic interaction between *MSH2*/*msh2* and *SENP6*/ *ulp2* is due to the combined loss of MutSβ- and RPA-mediated DNA DSB repair. Consistent with this notion, the addition of Olaparib (a PARP inhibitor that induces the accumulation of ssDNA breaks that ultimately result in DSBs during replication [99-101]) rendered a significantly greater number of γH2AX^+^ cells in the (*MSH2*/*SENP6*) deficient population versus singly-deficient controls (Figure 5F). This is accompanied by the reduced clonogenic survival of *MSH2*^*−*^ cells in response to *SENP6* knockdown, olaparib treatment, or their combination (Figure 5F). In isolation this synthetic interaction provides no direct mechanistic insight, but further analyses showed that the (*MSH2*/*SENP6*) deficient population also displayed a hyper-acute apoptotic response to olaparib (Figure 5H). It appears likely that (*MSH2*/*SENP6*) deficient cells are incapable of resolving the genomic lesions induced by PARP-inhibition, with apoptosis becoming a preferred response. Olaparib is currently in phase II clinical trials for the treatment of *BRCA1/2*-dependent breast and ovarian cancers [102-104]. Our findings suggest that it may also enhance the efficacy of strategies that target components of DSB repair pathways in MSH2-deficient tumors.

## Conclusions

This study describes the identification of conserved genetic interactions between the MutSβ subunits and orthologous genes encoding diverse biological functions in two evolutionarily distant yeast species. It further demonstrates that unexpected genetic relationships could be exploited to treat loss-of-function disorders. In the specific example presented, the synthetic interaction of (*MSH2*/*SENP6*) deficiency enhanced by PARP inhibitors indicates that a significant proportion of MMR-deficient cancers could be targeted via their loss of function in MutSβ-mediated recombinatorial, rather than mismatch, repair [10] (Figure 1A). Ongoing studies have established the central role of reversible sumoylation in an efficient DNA damage response, suggesting that the SUMO pathway might contain novel druggable targets for anticancer therapeutics [79,105]. In this work we suggest that targeting specific SUMO proteases may be highly effective in the treatment of MMR-deficient cancers. The recent development of SENP6-specific small molecule inhibitors [106] could prove invaluable for further experiments in this regard.

## Additional file

Additional file 1:
**Additional information is available with the online version of this paper.** This single file contains all siRNA sequences, data regarding the specificity of anti-SENP6 (Figure S1) and detailed tetrad data analyzing the genetic relationship between *msh2*Δ and deletions of the fission yeast protein sumoylation machinery (Figure S2).

## Abbreviations

5-FU: 5-fluorouracil
CGI: Conserved genetic interaction
CRC: Colorectal cancer
DSB: Double-strand break
GI: Genetic interaction
HNPCC/LS: Hereditary non-polyposis colorectal cancer/Lynch syndrome
HR: Homologous recombination
MMR: DNA mismatch repair
*MSH*: MutS homolog
MSI: Microsatellite instability
RNAi: RNA interference
*Sc*: *Saccharomyces cerevisiae*
*Sp*: *Schizosaccharomyces pombe*
SS/SL: Synthetic sick/synthetic lethal

## References

[1] Rustgi AK (2007). The genetics of hereditary colon cancer. Genes Dev.

[2] Hampel H, Frankel WL, Martin E, Arnold M, Khanduja K, Kuebler P, Nakagawa H, Sotamaa K, Prior TW, Westman J, Panescu J, Fix D, Lockman J, Comeras I, de la Chapelle A (2005). Screening for the Lynch syndrome (hereditary nonpolyposis colorectal cancer). N Engl J Med.

[3] Iyer RR, Pluciennik A, Burdett V, Modrich PL (2006). DNA mismatch repair: functions and mechanisms. Chem Rev.

[4] Kunkel TA, Erie DA (2005). DNA mismatch repair. Annu Rev Plant Physiol Plant Mol Biol.

[5] Harrington JM, Kolodner RD (2007). Saccharomyces cerevisiae Msh2-Msh3 acts in repair of base-base mispairs. Mol Cell Biol.

[6] Acharya S, Wilson T, Gradia S, Kane MF, Guerrette S, Marsischky GT, Kolodner R, Fishel R (1996). hMSH2 forms specific mispair-binding complexes with hMSH3 and hMSH6. Proc Natl Acad Sci U S A.

[7] Srivatsan A, Bowen N, Kolodner RD (2014). Mispair-specific recruitment of the Mlh1-Pms1 complex identifies repair substrates of the *Saccharomyces cerevisiae* Msh2-Msh3 complex. J Biol Chem.

[8] Palombo F, Iaccarino I, Nakajima E, Ikejima M, Shimada T, Jiricny J (1996). hMutSbeta, a heterodimer of hMSH2 and hMSH3, binds to insertion/deletion loops in DNA. Curr Biol.

[9] Umar A, Risinger JI, Glaab WE, Tindall KR, Barrett JC, Kunkel TA (1998). Functional overlap in mismatch repair by human MSH3 and MSH6. Genetics.

[10] van Oers JM, Edwards Y, Chahwan R, Zhang W, Smith C, Pechuan X, Schaetzlein S, Jin B, Wang Y, Bergman A, Scharff MD, Edelmann W (2013). The MutSβ complex is a modulator of p53-driven tumorigenesis through its functions in both DNA double-strand break repair and mismatch repair. Oncogene.

[11] Sugawara N, Pâques F, Colaiácovo M, Haber JE (1997). Role of Saccharomyces cerevisiae Msh2 and Msh3 repair proteins in double-strand break-induced recombination. Proc Natl Acad Sci U S A.

[12] Fink D, Aebi S, Howell SB (1998). The role of DNA mismatch repair in drug resistance. Clin Cancer Res.

[13] Jover R, Zapater P, Castells A, Llor X, Andreu M, Cubiella J, Balaguer F, Sempere L, Xicola RM, Bujanda L, Reñé JM, Clofent J, Bessa X, Morillas JD, Nicolás-Pérez D, Pons E, Payá A, Alenda C, Gastrointestinal Oncology Group of the Spanish Gastroenterological Association (2009). The efficacy of adjuvant chemotherapy with 5-fluorouracil in colorectal cancer depends on the mismatch repair status. Eur J Cancer.

[14] Mao G, Yuan F, Absher K, Jennings CD, Howard DS, Jordan CT, Gu L (2008). Preferential loss of mismatch repair function in refractory and relapsed acute myeloid leukemia: potential contribution to AML progression. Cell Res.

[15] Offman J, Opelz G, Doehler B, Cummins D, Halil O, Banner NR, Burke MM, Sullivan D, Macpherson P, Karran P (2004). Defective DNA mismatch repair in acute myeloid leukemia/myelodysplastic syndrome after organ transplantation. Blood.

[16] Bignami M, Casorelli I, Karran P (2003). Mismatch repair and response to DNA-damaging antitumour therapies. Eur J Cancer.

[17] Kaelin WG (2005). The concept of synthetic lethality in the context of anti-cancer therapy. Nat Rev Cancer.

[18] Chan DA, Giaccia AJ (2011). Harnessing synthetic lethal interactions in anticancer drug discovery. Nat Rev Drug Discov.

[19] Farmer H, McCabe N, Lord CJ, Tutt AN, Johnson DA, Richardson TB, Santarosa M, Dillon KJ, Hickson I, Knights C, Martin NM, Jackson SP, Smith GC, Ashworth A (2005). Targeting the DNA repair defect in BRCA mutant cells as a therapeutic strategy. Nature.

[20] Hay T, Matthews JR, Pietzka L, Lau A, Cranston A, Nygren AO, Douglas-Jones A, Smith GC, Martin NM, O’Connor M, Clarke AR (2009). Poly(ADP-ribose) polymerase-1 inhibitor treatment regresses autochthonous Brca2/p53-mutant mammary tumors in vivo and delays tumor relapse in combination with carboplatin. Cancer Res.

[21] Roguev A, Talbot D, Negri GL, Shales M, Cagney G, Bandyopadhyay S, Panning B, Krogan NJ (2013). Quantitative genetic-interaction mapping in mammalian cells. Nat Methods.

[22] Iorns E, Lord CJ, Turner N, Ashworth A (2007). Utilizing RNA interference to enhance cancer drug discovery. Nat Rev Drug Discov.

[23] Luo J, Emanuele MJ, Li D, Creighton CJ, Schlabach MR, Westbrook TF, Wong KK, Elledge SJ (2009). A genome-wide RNAi screen identifies multiple synthetic lethal interactions with the Ras oncogene. Cell.

[24] Kim YW, Liu T, Koul D, Tiao N, Feroze AH, Wang J, Powis G, Yung WK (2011). Identification of novel synergistic targets for rational drug combinations with PI3 kinase inhibitors using siRNA synthetic lethality screening against GBM. Neuro Oncol.

[25] Martin SA, McCabe N, Mullarkey M, Cummins R, Burgess DJ, Nakabeppu Y, Oka S, Kay E, Lord CJ, Ashworth A (2010). DNA polymerases as potential therapeutic targets for cancers deficient in the DNA mismatch repair proteins MSH2 or MLH1. Cancer Cell.

[26] Martin SA, McCarthy A, Barber LJ, Burgess DJ, Parry S, Lord CJ, Ashworth A (2009). Methotrexate induces oxidative DNA damage and is selectively lethal to tumour cells with defects in the DNA mismatch repair gene MSH2. EMBO Mol Med.

[27] Loeb LA, Monnat RJ (2008). DNA polymerases and human disease. Nat Rev Genet.

[28] Cabelof DC, Ikeno Y, Nyska A, Busuttil RA, Anyangwe N, Vijg J, Matherly LH, Tucker JD, Wilson SH, Richardson A, Heydari AR (2006). Haploinsufficiency in DNA polymerase beta increases cancer risk with age and alters mortality rate. Cancer Res.

[29] Boone C, Bussey H, Andrews BJ (2007). Exploring genetic interactions and networks with yeast. Nat Rev Genet.

[30] McGary KL, Park TJ, Woods JO, Cha HJ, Wallingford JB, Marcotte EM (2010). Systematic discovery of nonobvious human disease models through orthologous phenotypes. Proc Natl Acad Sci U S A.

[31] Sipiczki M (2000). Where does fission yeast sit on the tree of life?. Genome Biol.

[32] O’Brien SJ, Menotti-Raymond M, Murphy WJ, Nash WG, Wienberg J, Stanyon R, Copeland NJ, Jenkins NA, Womack JE, Graves JA (1999). The promise of comparative genomics in mammals. Science.

[33] Dixon SJ, Fedyshyn Y, Koh JL, Prasad TS, Chahwan C, Chua G, Toufighi K, Baryshnikova A, Hayles J, Hoe KL, Kim DU, Park HO, Myers CL, Pandey A, Durocher D, Andrews BJ, Boone C (2008). Significant conservation of synthetic lethal genetic interaction networks between distantly related eukaryotes. Proc Natl Acad Sci U S A.

[34] Roguev A, Bandyopadhyay S, Zofall M, Zhang K, Fischer T, Collins SR, Qu H, Shales M, Park HO, Hayles J, Hoe KL, Kim DU, Ideker T, Grewal SI, Weissman JS, Krogan NJ (2008). Conservation and rewiring of functional modules revealed by an epistasis map (E-MAP) in fission yeast. Science.

[35] Kim DU, Hayles J, Kim D, Wood V, Park HO, Won M, Yoo HS, Duhig T, Nam M, Palmer G, Han S, Jeffery L, Baek ST, Lee H, Shim YS, Lee M, Kim L, Heo KS, Noh EJ, Lee AR, Jang YJ, Chung KS, Choi SJ, Park JY, Park Y, Kim HM, Park SK, Park HJ, Kang EJ, Kim HB (2010). Analysis of a genome-wide set of gene deletions in the fission yeast *Schizosaccharomyces pombe*. Nat Biotechnol.

[36] Shevchenko A, Roguev A, Schaft D, Buchanan L, Habermann B, Sakalar C, Thomas H, Krogan NJ, Shevchenko A, Stewart AF (2008). Chromatin Central: towards the comparative proteome by accurate mapping of the yeast proteomic environment. Genome Biol.

[37] Bao Y, Shen X (2007). SnapShot: chromatin remodeling complexes. Cell.

[38] Tong AH, Lesage G, Bader GD, Ding H, Xu H, Xin X, Young J, Berriz GF, Brost RL, Chang M, Chen Y, Cheng X, Chua G, Friesen H, Goldberg DS, Haynes J, Humphries C, He G, Hussein S, Ke L, Krogan N, Li Z, Levinson JN, Lu H, Ménard P, Munyana C, Parsons AB, Ryan O, Tonikian R, Roberts T (2004). Global mapping of the yeast genetic interaction network. Science.

[39] Giaever G, Chu AM, Ni L, Connelly C, Riles L, Veronneau S, Dow S, Lucau-Danila A, Anderson K, André B, Arkin AP, Astromoff A, El-Bakkoury M, Bangham R, Benito R, Brachat S, Campanaro S, Curtiss M, Davis K, Deutschbauer A, Entian KD, Flaherty P, Foury F, Garfinkel DJ, Gerstein M, Gotte D, Güldener U, Hegemann JH, Hempel S, Herman Z (2002). Functional profiling of the *Saccharomyces cerevisiae* genome. Nature.

[40] Costanzo M, Baryshnikova A, Bellay J, Kim Y, Spear ED, Sevier CS, Ding H, Koh JL, Toufighi K, Mostafavi S, Prinz J, St Onge RP, VanderSluis B, Makhnevych T, Vizeacoumar FJ, Alizadeh S, Bahr S, Brost RL, Chen Y, Cokol M, Deshpande R, Li Z, Lin ZY, Liang W, Marback M, Paw J, San Luis BJ, Shuteriqi E, Tong AH, van Dyk N (2010). The genetic landscape of a cell. Science.

[41] Frost A, Elgort MG, Brandman O, Ives C, Collins SR, Miller-Vedam L, Weibezahn J, Hein MY, Poser I, Mann M, Hyman AA, Weissman JS (2012). Functional repurposing revealed by comparing *S. pombe* and *S. cerevisiae* genetic interactions. Cell.

[42] Ryan CJ, Roguev A, Patrick K, Xu J, Jahari H, Tong Z, Beltrao P, Shales M, Qu H, Collins SR, Kliegman JI, Jiang L, Kuo D, Tosti E, Kim HS, Edelmann W, Keogh MC, Greene D, Tang C, Cunningham P, Shokat KM, Cagney G, Svensson JP, Guthrie C, Espenshade PJ, Ideker T, Krogan NJ (2012). Hierarchical modularity and the evolution of genetic interactomes across species. Mol Cell.

[43] Modrich PL (2006). Mechanisms in eukaryotic mismatch repair. J Biol Chem.

[44] Keogh M-C, Cho E-J, Podolny V, Buratowski S (2002). Kin28 is found within TFIIH and a Kin28-Ccl1-Tfb3 trimer complex with differential sensitivities to T-loop phosphorylation. Mol Cell Biol.

[45] Janke C, Magiera MM, Rathfelder N, Taxis C, Reber S, Maekawa H, Moreno-Borchart A, Doenges G, Schwob E, Schiebel E, Knop M (2004). A versatile toolbox for PCR-based tagging of yeast genes: new fluorescent proteins, more markers and promoter-substitution cassettes. Yeast.

[46] Kim H-S, Vanoosthuyse V, Fillingham J, Roguev A, Watt S, Kislinger T, Treyer A, Carpenter LR, Bennett CS, Emili A, Greenblatt JF, Hardwick KG, Krogan NJ, Bähler J, Keogh MC (2009). An acetylated form of histone H2A.Z regulates chromosome architecture in *Schizosaccharomyces pombe*. Nat Struct Mol Biol.

[47] Roguev A, Wiren M, Weissman JS, Krogan NJ (2007). High-throughput genetic interaction mapping in the fission yeast *Schizosaccharomyces pombe*. Nat Methods.

[48] Li GM (2008). Mechanisms and functions of DNA mismatch repair. Cell Res.

[49] Watanabe Y, Haugen-Strano A, Umar A, Yamada K, Hemmi H, Kikuchi Y, Takano S, Shibata Y, Barrett JC, Kunkel TA, Koi M (2000). Complementation of an hMSH2 defect in human colorectal carcinoma cells by human chromosome 2 transfer. Mol Carcinog.

[50] Palliser D, Chowdhury D, Wang QY, Lee SJ, Bronson RT, Knipe DM, Lieberman J (2006). An siRNA-based microbicide protects mice from lethal herpes simplex virus 2 infection. Nature.

[51] Edelmann W, Umar A, Yang K, Heyer J, Kucherlapati M, Lia M, Kneitz B, Avdievich E, Fan K, Wong E, Crouse G, Kunkel T, Lipkin M, Kolodner RD, Kucherlapati R (2000). The DNA mismatch repair genes Msh3 and Msh6 cooperate in intestinal tumor suppression. Cancer Res.

[52] Kim TM, Laird PW, Park PJ (2013). The landscape of microsatellite instability in colorectal and endometrial cancer genomes. Cell.

[53] Plaschke J, Kruger S, Jeske B, Theissig F, Kreuz FR, Pistorius S, Saeger HD, Iaccarino I, Marra G, Schackert HK (2004). Loss of MSH3 protein expression is frequent in MLH1-deficient colorectal cancer and is associated with disease progression. Cancer Res.

[54] Poulogiannis G, Frayling IM, Arends MJ (2010). DNA mismatch repair deficiency in sporadic colorectal cancer and Lynch syndrome. Histopathology.

[55] Marischky GT, Foliso N, Kane MF, Kolodner R (1996). Redundancy of *Saccharomyces cerevisiae MSH3* and *MSH6* in *MSH2*-dependent mismatch repair. Genes Dev.

[56] Mansour AA, Tornier C, Lehmann E, Darmon M, Fleck O (2001). Control of GT repeat stability in Schizosaccharomyces pombe by mismatch repair factors. Genetics.

[57] Fishel R, Lescoe MK, Rao MR, Copeland NG, Jenkins NA, Garber J, Kane M, Kolodner R (1993). The human mutator gene homolog MSH2 and its association with hereditary nonpolyposis colon cancer. Cell.

[58] Rudolph C, Kunz C, Parisi S, Lehmann E, Hartsuiker E, Fartmann B, Kramer W, Kohli J, Fleck O (1999). The msh2 gene of Schizosaccharomyces pombe is involved in mismatch repair, mating-type switching, and meiotic chromosome organization. Mol Cell Biol.

[59] Collins SR, Miller KM, Maas NL, Roguev A, Fillingham J, Chu CS, Schuldiner M, Gebbia M, Recht J, Shales M, Ding H, Xu H, Han J, Ingvarsdottir K, Cheng B, Andrews B, Boone C, Berger SL, Hieter P, Zhang Z, Brown GW, Ingles CJ, Emili A, Allis CD, Toczyski DP, Weissman JS, Greenblatt JF, Krogan NJ (2007). Functional dissection of protein complexes involved in yeast chromosome biology using a genetic interaction map. Nature.

[60] Schuldiner M, Collins SR, Weissman JS, Krogan NJ (2006). Quantitative genetic analysis in *Saccharomyces cerevisiae* using epistatic miniarray profiles (E-MAPs) and its application to chromatin functions. Methods.

[61] Bandyopadhyay S, Mehta M, Kuo D, Sung M-K, Chuang R, Jaehnig EJ, Bodenmiller B, Licon K, Copeland W, Shales M, Fiedler D, Dutkowski J, Guénolé A, van Attikum H, Shokat KM, Kolodner RD, Huh WK, Aebersold R, Keogh MC, Krogan NJ, Ideker T (2010). Rewiring of genetic networks in response to DNA damage. Science.

[62] Raji H, Hartsuiker E (2006). Double-strand break repair and homologous recombination in *Schizosaccharomyces pombe*. Yeast.

[63] Sun W, Nandi S, Osman F, Ahn JS, Jakovleska J, Lorenz A, Whitby MC (2008). The FANCM ortholog Fml1 promotes recombination at stalled replication forks and limits crossing over during DNA double-strand break repair. Mol Cell.

[64] Saparbaev M, Prakash L, Prakash S (1996). Requirement of mismatch repair genes MSH2 and MSH3 in the RAD1-RAD10 pathway of mitotic recombination in *Saccharomyces cerevisiae*. Genetics.

[65] Nicholson A, Fabbri RM, Reeves JW, Crouse GF (2006). The effects of mismatch repair and RAD1 genes on interchromosomal crossover recombination in *Saccharomyces cerevisiae*. Genetics.

[66] Guénolé A, Srivas R, Vreeken K, Wang ZZ, Wang S, Krogan NJ, Ideker T, van Attikum H (2013). Dissection of DNA damage responses using multiconditional genetic interaction maps. Mol Cell.

[67] Reinke A, Anderson S, McCaffery JM, Yates J, Aronova S, Chu S, Fairclough S, Iverson C, Wedaman KP, Powers T (2004). TOR complex 1 includes a novel component, Tco89p (YPL180w), and cooperates with Ssd1p to maintain cellular integrity in *Saccharomyces cerevisiae*. J Biol Chem.

[68] Toda T, Cameron S, Sass P, Wigler M (1988). SCH9, a gene of *Saccharomyces cerevisiae* that encodes a protein distinct from, but functionally and structurally related to, cAMP-dependent protein kinase catalytic subunits. Genes Dev.

[69] Rout MP, Aitchison JD, Suprapto A, Hjertaas K, Zhao Y, Chait BT (2000). The yeast nuclear pore complex: composition, architecture, and transport mechanism. J Cell Biol.

[70] Saravanan M, Wuerges J, Bose D, McCormack EA, Cook NJ, Zhang X, Wigley DB (2012). Interactions between the nucleosome histone core and Arp8 in the INO80 chromatin remodeling complex. Proc Natl Acad Sci U S A.

[71] Harnpicharnchai P, Jakovljevic J, Horsey E, Miles T, Roman J, Rout M, Meagher D, Imai B, Guo Y, Brame CJ, Shabanowitz J, Hunt DF, Woolford JL (2001). Composition and functional characterization of yeast 66S ribosome assembly intermediates. Mol Cell.

[72] Vilar E, Mukherjee B, Kuick R, Raskin L, Misek DE, Taylor JM, Giordano TJ, Hanash SM, Fearon ER, Rennert G, Gruber SB (2009). Gene expression patterns in mismatch repair-deficient colorectal cancers highlight the potential therapeutic role of inhibitors of the phosphatidylinositol 3-kinase-AKT-mammalian target of rapamycin pathway. Clin Cancer Res.

[73] Palancade B, Liu X, Garcia-Rubio M, Aguilera A, Zhao X, Doye V (2007). Nucleoporins prevent DNA damage accumulation by modulating Ulp1-dependent sumoylation processes. Mol Biol Cell.

[74] Parsons AB, Brost RL, Ding H, Li Z, Zhang CL, Sheikh B, Brown GW, Kane PM, Hughes TR, Boone C (2004). Integration of chemical-genetic and genetic interaction data links bioactive compounds to cellular target pathways. Nat Biotechnol.

[75] Li SJ, Hochstrasser M (2000). The yeast ULP2 (SMT4) gene encodes a novel protease specific for the ubiquitin-like Smt3 protein. Mol Cell Biol.

[76] Bylebyl GR, Belichenko I, Johnson ES (2003). The SUMO isopeptidase Ulp2 prevents accumulation of SUMO chains in yeast. J Biol Chem.

[77] Uzunova K, Gottsche K, Miteva M, Weisshaar SR, Glanemann C, Schnellhardt M, Niessen M, Scheel H, Hofmann K, Johnson ES, Praefcke GJ, Dohmen RJ (2007). Ubiquitin-dependent proteolytic control of SUMO conjugates. J Biol Chem.

[78] Dziadkowiec D, Petters E, Dyjankiewicz A, Karpiński P, Garcia V, Watson A, Carr AM (2009). The role of novel genes rrp1(+) and rrp2(+) in the repair of DNA damage in *Schizosaccharomyces pombe*. DNA Repair (Amst).

[79] Jackson SP, Durocher D (2013). Regulation of DNA Damage Responses by Ubiquitin and SUMO. Mol Cell.

[80] Cremona CA, Sarangi P, Yang Y, Hang LE, Rahman S, Zhao X (2012). Extensive DNA damage-induced sumoylation contributes to replication and repair and acts in addition to the mec1 checkpoint. Mol Cell.

[81] Nagai S, Davoodi N, Gasser SM (2011). Nuclear organization in genome stability: SUMO connections. Cell Res.

[82] Watts FZ, Skilton A, Ho JC, Boyd LK, Trickey MA, Gardner L, Ogi FX, Outwin EA (2007). The role of *Schizosaccharomyces pombe* SUMO ligases in genome stability. Biochem Soc Trans.

[83] Stephan AK, Kliszczak M, Morrison CG (2011). The Nse2/Mms21 SUMO ligase of the Smc5/6 complex in the maintenance of genome stability. FEBS Lett.

[84] Elmore ZC, Donaher M, Matson BC, Murphy H, Westerbeck JW, Kerscher O (2011). Sumo-dependent substrate targeting of the SUMO protease Ulp1. BMC Biol.

[85] Soustelle C, Vernis L, Fréon K, Reynaud-Angelin A, Chanet R, Fabre F, Heude M (2004). A new *Saccharomyces cerevisiae* strain with a mutant Smt3-deconjugating Ulp1 protein is affected in DNA replication and requires Srs2 and homologous recombination for its viability. Mol Cell Biol.

[86] Bekes M, Prudden J, Srikumar T, Raught B, Boddy MN, Salvesen GS (2011). The dynamics and mechanism of SUMO chain deconjugation by SUMO-specific proteases. J Biol Chem.

[87] Felberbaum R, Hochstrasser M (2008). Ulp2 and the DNA damage response: desumoylation enables safe passage through mitosis. Cell Cycle.

[88] Lee MT, Bakir AA, Nguyen KN, Bachant J (2011). The SUMO isopeptidase Ulp2p is required to prevent recombination-induced chromosome segregation lethality following DNA replication stress. PLoS Genet.

[89] McDonald WH, Pavlova Y, Yates JR, Boddy MN (2003). Novel essential DNA repair proteins Nse1 and Nse2 are subunits of the fission yeast Smc5-Smc6 complex. J Biol Chem.

[90] Mukhopadhyay D, Dasso M (2007). Modification in reverse: the SUMO proteases. Trends Biochem Sci.

[91] Kolli N, Mikolajczyk J, Drag M, Mukhopadhyay D, Moffatt N, Dasso M, Salvesen G, Wilkinson KD (2010). Distribution and paralogue specificity of mammalian deSUMOylating enzymes. Biochem J.

[92] Alegre KO, Reverter D (2011). Swapping small ubiquitin-like modifier (SUMO) isoform specificity of SUMO proteases SENP6 and SENP7. J Biol Chem.

[93] Dou H, Huang C, Singh M, Carpenter PB, Yeh ET (2010). Regulation of DNA repair through deSUMOylation and SUMOylation of replication protein A complex. Mol Cell.

[94] Maison C, Romeo K, Bailly D, Dubarry M, Quivy JP, Almouzni G (2012). The SUMO protease SENP7 is a critical component to ensure HP1 enrichment at pericentric heterochromatin. Nat Struct Mol Biol.

[95] Bawa-Khalfe T, Lu LS, Zuo Y, Huang C, Dere R, Lin FM, Yeh ET (2012). Differential expression of SUMO-specific protease 7 variants regulates epithelial-mesenchymal transition. Proc Natl Acad Sci U S A.

[96] Garvin AJ, Densham RM, Blair-Reid SA, Pratt KM, Stone HR, Weekes D, Lawrence KJ, Morris JR (2013). The deSUMOylase SENP7 promotes chromatin relaxation for homologous recombination DNA repair. EMBO Rep.

[97] Rogakou EP, Pilch DR, Orr AH VSI, Bonner WP (1998). DNA double-strand breaks induce H2AX phosphorylation on Serine 139. J Biol Chem.

[98] Takahashi M, Koi M, Balaguer F, Boland CR, Goel A (2011). MSH3 mediates sensitization of colorectal cancer cells to cisplatin, oxaliplatin, and a poly(ADP-ribose) polymerase inhibitor. J Biol Chem.

[99] Saffhill R, Ockey CH (1985). Strand breaks arising from the repair of the 5-bromodeoxyuridine-substituted template and methyl methanesulphonate-induced lesions can explain the formation of sister chromatid exchanges. Chromosoma.

[100] Rouleau M, Patel A, Hendzel MJ, Kaufmann SH, Poirier GG (2010). PARP inhibition: PARP1 and beyond. Nat Rev Cancer.

[101] Zaremba T, Curtin NJ (2007). PARP inhibitor development for systemic cancer targeting. Anticancer Agents Med Chem.

[102] Bryant HE, Schultz N, Thomas HD, Parker KM, Flower D, Lopez E, Kyle S, Meuth M, Curtin NJ, Helleday T (2005). Specific killing of BRCA2-deficient tumours with inhibitors of poly(ADP-ribose) polymerase. Nature.

[103] Tutt A, Robson M, Garber JE, Domchek SM, Audeh MW, Weitzel JN, Friedlander M, Arun B, Loman N, Schmutzler RK, Wardley A, Mitchell G, Earl H, Wickens M, Carmichael J (2010). Oral poly(ADP-ribose) polymerase inhibitor olaparib in patients with BRCA1 or BRCA2 mutations and advanced breast cancer: a proof-of-concept trial. Lancet.

[104] Ang JE, Gourley C, Powell CB, High H, Shapira-Frommer R, Castonguay V, De Greve J, Atkinson T, Yap TA, Sandhu S, Banerjee S, Chen LM, Friedlander ML, Kaufman B, Oza AM, Matulonis U, Barber LJ, Kozarewa I, Fenwick K, Assiotis I, Campbell J, Chen L, de Bono JS, Gore ME, Lord CJ, Ashworth A, Kaye SB (2013). Efficacy of chemotherapy in BRCA1/2 mutation carrier ovarian cancer in the setting of PARP inhibitor resistance: a multi-institutional study. Clin Cancer Res.

[105] Qian J, Luo Y, Gu X, Wang X (2013). Inhibition of SENP6-induced radiosensitization of human hepatocellular carcinoma cells by blocking radiation-induced NF-κB activation. Cancer Biother Radiopharm.

[106] Albrow VE, Ponder EL, Fasci D, Békés M, Deu E, Salvesen GS, Bogyo M (2011). Development of small molecule inhibitors and probes of human SUMO deconjugating proteases. Chem Biol.

