# Supplementary material for: Evolutionarily conserved genetic interactions with budding and fission yeast MutS identify orthologous relationships in mismatch repair-deficient cancer cells
Source: Genome Med. 2014 Sep 17;6:68. doi: 10.1186/s13073-014-0068-4 (PMC4189729; doi:10.1186/s13073-014-0068-4)
Supplement: Additional file 1: — Additional information is available with the online version of this paper. This single file contains all siRNA sequences, data regarding the specificity of anti-SENP6 (Figure S1) and detailed tetrad data analyzing the genetic relationship between msh2Δ and deletions of the fission yeast protein sumoylation machinery (Figure S2). [file 13073_2014_68_MOESM1_ESM.pdf]

Evolutionarily conserved genetic interactions with budding and fission yeast *MutS* identify orthologous relationships in mismatch repair-deficient cancer cells

Elena Tosti [1], Joseph A. Katakowski [2], Sonja Schaetzlein [1], Hyun-Soo Kim [1], Colm J. Ryan [3, 4, 5], Michael Shales [3], Assen Roguev [3], Nevan J. Krogan [3, 4, 6], Deborah Palliser [2], Michael-Christopher Keogh [1, *] & Winfried Edelmann [1, *]

[1] Dept. of Cell Biology, Albert Einstein College of Medicine, New York, USA

[2] Dept. of Microbiology & Immunology, Albert Einstein College of Medicine, New York, USA

[3] Dept. of Cellular & Molecular Pharmacology, UCSF, San Francisco, USA

[4] California Institute for Quantitative Biosciences, San Francisco, USA

[5] School of Medicine and Medical Science, University College Dublin, Ireland

[6] J. David Gladstone Institutes, San Francisco, USA

* To whom correspondence should be addressed -

Email: michael.keogh@einstein.yu.edu, winfried.edelmann@einstein.yu.edu

Tel: 718 430 1086 Fax: 718 430 8574

Supplementary Information

2 x Supplementary Figures + Legends

Supplementary References

SUPPLEMENTARY INFORMATION

siRNA sequences - *HEC59* cancer cells (*MSH2*$^{-}$) and their isogenic chromosome 2-complemented counterparts (*MSH2*$^{+}$) [1] in six-well plates were transfected with siRNAs (*Dharmacon*) and *Trans*IT-siQUEST (*Mirus*) by specific optimization of the manufacturers suggested conditions. Each targeted siRNA gave a similar result (with regard to knockdown and phenotypic outcome) when used individually or appropriately pooled, reducing the likelihood of off-target effects.

siSENP6-1 (sense strand): GGACCAAUCUGCUCAGUGU [2]

siSENP6-C (sense strand): GCACAGAUACCAGUAGUAA

siSENP7-1 (sense strand): GGAAAGAAGCCUAAGGAAU

siSENP7-2 (sense strand): GAGAAGAAUUGAAGCUGAA

siNT-1 (sense strand): UAGCGACUAAACACAUCAA

SUPPLEMENTARY FIGURE LEGENDS

Figure S1 - Specificity of anti-SENP6. *SENP6* protein levels in permeabilized *HEC59* cells (*MSH2*$^{-}$) were measured by fluorescence cytometry. In brief, cells were trypsinized, stained with *LIVE/DEAD fixable violet* (*Invitrogen*), fixed with 2% paraformaldehyde, and permeabilized as noted. Each sample was then successively stained with monoclonal mouse anti-human SENP6 (αSENP6; *Novus Biologicals*) or an IgG2a-isotype control (*eBioscience*) and anti-mouse IgG2a-PE (2nd Ab; *eBioscience*), and analyzed by fluorescence cytometry. **(A)** Optimization of permeabilization approach (0.3% Saponin vs. 0.1% Triton X-100). **(B)** Titration of monoclonal mouse anti-human SENP6 (*, 1:400 chosen) after permeabilization with 0.3% Saponin.

Figure S2 - Polysumoylation is lethal in *msh2Δ* fission yeast. Strains were mated, sporulated, and the indicated number of tetrads dissected. The number of viable spores observed / expected (assuming independent Mendelian segregation) for each genotype is indicated. **(B-C)** *msh2Δ* is synthetic lethal with *ulp2Δ* but shows no genetic interaction with *ulp1Δ*. **(D-F)** *smt3Δ* or *nse2-SA* (catalytic dead *nse2-C195S, H197A*), but not *pli1Δ*, rescue the lethality of [*msh2Δ* / *ulp2Δ*]. ** Msh2 and Smt3 are genetically linked (separated by only 159,352bp on chromosome I), explaining the unexpectedly large number of triple mutant spores.

SUPPLEMENTARY REFERENCES

1. Watanabe Y, Haugen-Strano A, Umar A, Yamada K, Hemmi H, Kikuchi Y, Takano S, Shibata Y, Barrett JC, Kunkel TA, Koi M: **Complementation of an hMSH2 defect in human colorectal carcinoma cells by human chromosome 2 transfer.** *Mol Carcinog* 2000, **29:**37-49.
2. Dou H, Huang C, Singh M, Carpenter PB, Yeh ET: **Regulation of DNA repair through deSUMOylation and SUMOylation of replication protein A complex.** *Mol Cell* 2010, **39:**333-345.

Figure S1: Tosti *et al*

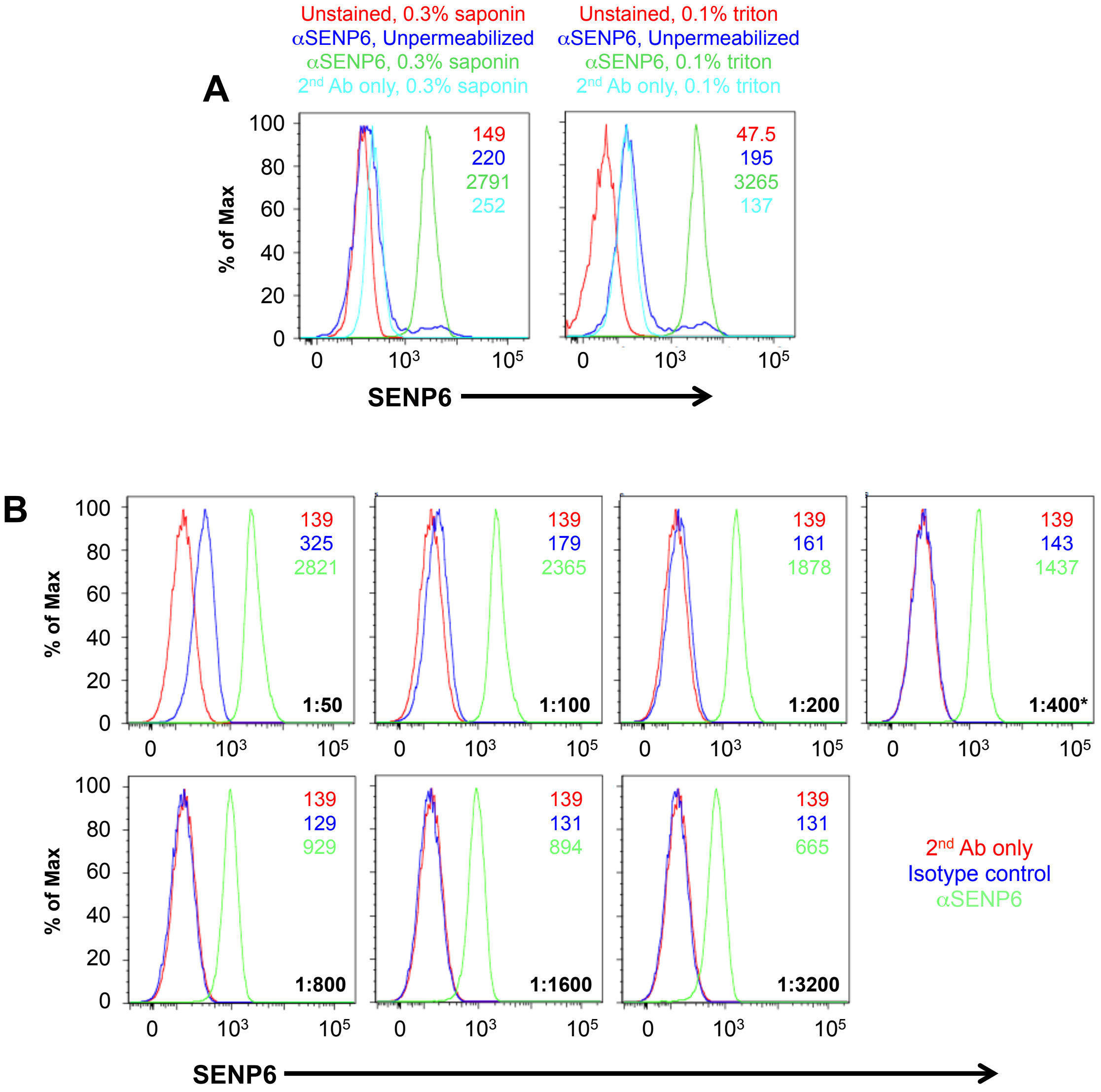

Figure S2: Tosti *et al*

A

Genotype	# viable spores	% observed	% expected
WT	12	27.3	25
msh2Δ	10	22.7	25
ulp1Δ	10	22.7	25
msh2Δ / *ulp1Δ*	12	27.3	25
MISSING	0	0	0
**TOTAL**	**44**	**100**	**100**

B

Genotype	# viable spores	% observed	% expected
WT	39	26.4	25
msh2Δ	39	26.4	25
ulp2Δ	35	23.6	25
msh2Δ / *ulp2Δ*	1 *	0.7	25
MISSING	34	23	0
**TOTAL**	**148**	**100**	**100**

C

Genotype	# viable spores	% observed	% expected
WT	15	22.1	12.5
msh2Δ	3	4.4	12.5
smt3Δ	3	4.4	12.5
ulp2Δ	10	14.7	12.5
smt3Δ / *ulp2Δ*	1 *	1.5	12.5
smt3Δ / *msh2Δ*	10	14.7	12.5
msh2Δ / *ulp2Δ*	0	0	12.5
smt3Δ / msh2Δ / ulp2Δ	16 **	23.5	12.5
MISSING	10	14.7	0
**TOTAL**	**68**	**100**	**100**

D

Genotype	# viable spores	% observed	% expected
WT	22	20.4	12.5
msh2Δ	16	14.8	12.5
nse2-SA	12	11.1	12.5
ulp2Δ	12	11.1	12.5
nse2-SA / *ulp2Δ*	7	6.5	12.5
nse2-SA / *msh2Δ*	13	12	12.5
msh2Δ / *ulp2Δ*	0	0	12.5
nse2-SA / msh2Δ / ulp2Δ	13	12	12.5
MISSING	13	12	0
**TOTAL**	**108**	**100**	**100**

E

Genotype	# viable spores	% observed	% expected
WT	13	11.2	12.5
msh2Δ	20	17.2	12.5
pli1Δ	14	12.1	12.5
ulp2Δ	14	12.1	12.5
pli1Δ / *ulp2Δ*	12	10.3	12.5
pli1Δ / *msh2Δ*	10	8.6	12.5
msh2Δ / *ulp2Δ*	0	0	12.5
pli1Δ / msh2Δ / ulp2Δ	1	0.9	12.5
MISSING	32	27.6	0
**TOTAL**	**116**	**100**	**100**

Small colony *
Smt3 & Msh2 are genetically linked **
